# Toxic anterior segment syndrome-an updated review

**DOI:** 10.1186/s12886-018-0939-3

**Published:** 2018-10-25

**Authors:** Choul Yong Park, Jimmy K. Lee, Roy S. Chuck

**Affiliations:** 1 Department of Ophthalmology, Dongguk University, Ilsan Hospital, Goyang, South Korea; 2 Department of Ophthalmology and Visual Sciences, Montefiore Medical Center, Albert Einstein College of Medicine, Bronx, NY USA

**Keywords:** Cataract, Toxic, TASS, Anterior chamber, Inflammation

## Abstract

**Background:**

Toxic anterior segment syndrome (TASS) can be a rare complication of anterior segment surgery. Here we reviewed the most recent advances in the understanding of TASS.

**Methods:**

English articles related to TASS were retrieved from “PubMed” using the following keywords; “toxic anterior segment syndrome” or “TASS”. The authors of this paper reviewed all the retrieved literature and critical findings were summarized.

**Results:**

The onset of TASS can vary from hours to months. The clinical manifestations are also variable. The causes of TASS are broad and continue to expand and could not be elucidated in over half of the reported cases. Prompt and thorough investigation to explore the causes of TASS is critical. Surgeons should be fully aware and updated regarding possible etiologies and make ceaseless efforts to prevent TASS. This effort begins with establishing TASS prevention protocols and regularly training surgical staff. Proper cleaning of surgical instruments is critical and should follow the guidelines set by The American Society of Cataract and Refractive Surgery TASS Task Force. When TASS occurs, sharing information with other ophthalmologists and reporting new causes is crucial for the prevention of outbreaks.

**Conclusions:**

Anterior segment surgeons should be reminded that TASS is mostly preventable by the establishment of TASS prevention protocols, regular surgical staff training and thorough adherence to recommendations for cleaning and sterilizing intraocular surgical instruments.

## Background

Toxic anterior segment syndrome (TASS) is characterized by sterile postoperative inflammation of anterior segment after intraocular surgery [1, 2]. Although TASS most often occurs after cataract surgery, it has also been reported after keratoplasty and posterior segment surgeries [3–5]. The inflammation can be mild with a minimal cellular reaction or severe enough to cause marked cornea edema and hypopyon. The onset can be acute (within days) or delayed (after several months) [1, 6]. The overall incidence of TASS was found to be 0.22% in a large case series [7]. Additionally, a significant number of reported cases have occurred as clusters of outbreaks [7–11]. In cases of severe TASS, prompt control of inflammation is essential to prevent any permanent damage to delicate ocular structures such as the corneal endothelium, trabecular meshwork and macula. TASS frequently resembles the symptoms and signs of early postoperative bacterial endophthalmitis and therefore, makes accurate diagnosis challenging [1, 12]. While prompt initiation of oral and fortified topical antibiotics is key to the treatment of bacterial endophthalmitis, TASS usually does not respond to antibiotics and instead requires strong topical or systemic steroids for resolution. However, considering the potential detrimental and irreversible ophthalmic sequelae of bacterial endophthalmitis, most cases of unusual postoperative inflammation after cataract surgery are regarded as infectious endophthalmitis until proven otherwise.

When TASS is suspected, it is important to perform a thorough investigation to determine the causative agent. This investigation should include surgical instruments and disposable medical devices, e.g. ophthalmic viscoelastic agents, medications, surgical drapes, and sterilization systems. However, even thorough clinical and laboratory investigations sometimes fail to find the causative agent in many cases of TASS [9].

**Fig. 1:**
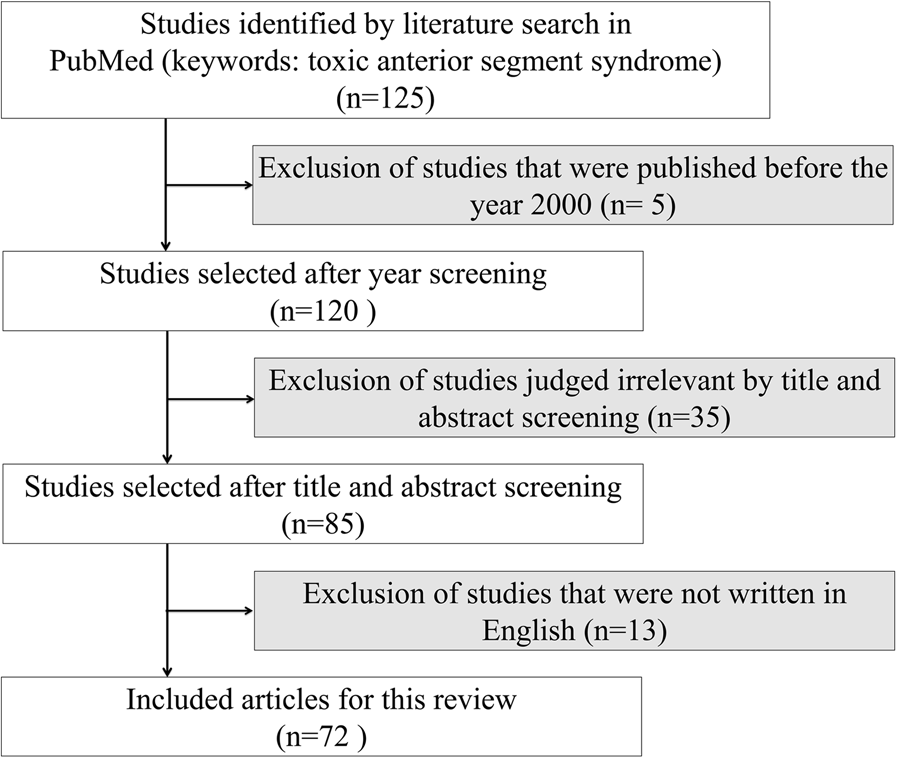
Flow chart to show the studies included in this review

By heightening professionals’ awareness and understanding of TASS, new causes of TASS are reported every year. This updated information should be shared amongst ophthalmic surgeons for effective prevention of TASS. With this report, we attempt to review recent advances in the understanding of TASS. Using the PubMed search engine, the keywords “toxic anterior segment” initially retrieved 125 articles. We excluded 5 articles published before the year 2000 and then screened the title and abstract of the remaining 120 articles. A further 35 articles were excluded from this review due to being irrelevant to our topic, and 13 non-English articles were excluded. Finally, 72 articles were included in this review (see Fig. 1).

### Clinical manifestations

TASS is typically characterized by unusual anterior chamber inflammation in the early postoperative period. Depending on the severity of inflammation, other symptoms may be present, such as pain, conjunctival injection or chemosis, hypopyon, corneal edema, keratic precipitates, anterior vitreous opacities, macular edema and visual deterioration [1, 6, 7, 13]. Most reported cases of TASS have been anecdotal, and therefore the clinical manifestations vary widely as shown in Table 1 [9, 10, 14–17]. However, in 2015 and 2017, two large case-series studies (*n* = 251 and *n* = 147) were conducted in Japan [8, 18]. The large sample sizes enabled the estimation of the occurrence rate of key clinical signs related to TASS (see Table 2). Anterior chamber reactions such as cell, flare and fibrin were the most common signs of TASS in these case series. Hypopyon, keratic precipitates and vitreous opacities were found in less than a quarter of cases.

**Table 1:** Summary of 15 recent case reports of toxic anterior segment syndrome

First Author (ref. no.)	Number of cases	Onset (days after the surgery)	Inciting agents	Clinical presentations	Managements (n)	Visual outcomes (n)
Miyake et al. [19]	6	42–167	IOL (ISert model 251, Hoya)	Chemosis, Ciliary injection, Decreased BCVA, Corneal edema, Anterior chamber reaction, hypopyon	Vitretomy and IOL removal (1), Capsule irrigation (2), Medical treatment only (5)	BCVA 20/100 (1), BCVA ≥20/30 (5)
Suzuki et al. [18]	251	38.44 ± 32.29 Range:0–161	IOL (ISert model 251 and 255, Hoya)	Anterior chamber reaction (99.2%), conjunctival injection (41.4%) Hypopyon (22.7%) Corneal edema (19.1%) Keratic precipitates (27.9%)	Medical treatment only (142), Surgical intervention: vitrectomy (49), IOL removal (22), chamber irrigation (51)	BCVA 0.036 ± 0.242 logMAR
Sorenson et al. [10]	10	1–7	Bacterial biofilm contamination of autoclave reservoir	Anterior chamber reaction (10), Hypopyon (3), Corneal edema (1), Anterior vitreous reaction (4)	Medical treatment only (3), Vitreous tap and intravitreal injection (7)	No light perception (1), BCVA≥20/30 (9)
Ohika et al. [8]	147	13.1 ± 16.4 Range: 1–88	IOL (Acrysof, Alcon)	Anterior chamber reaction (97.2%), Conjunctival injection (39.8%), Fibrinous inflammation (43.1%) Hypopyon (22.7%) Corneal edema (15.6%) Keratic precipitates (21.6%), Ocular pain (9.5%)	Medical treatment only (104), Surgical intervention: vitrectomy (21), IOL removal (10), chamber irrigation (33)	BCVA> 20/40 (143), BCVA≤20/40 (4)
Moyle et al. [9]	11	1	unknown	Corneal edema (11), anterior chamber reaction (10), Inflammatory plaque on IOL (5), hypopyon (3), fibrin reaction (6), mild pain (2)	Medical treatment only (11)	BCVA = 20/20 (11)
Sengupta et al. [7]	60	1	Balanced salt solution with a low pH of 6.0 (12), OVD (17), unknown (31)	Severe iridocyclitis with varying degree of corneal edema (60)	Medical treatment only (56), Vitreous tap (4)	BCVA: 0.11 ± 0.1 logMAR, range: 0–0.3 logMAR
Matsou et al. [36]	5	1	Generic trypan blue	Painless blurry vision, corneal edema, anterior chamber reaction, hypopyon and fibrin reaction	Medical treatment only (5)	BCVA: 0.82 ± 0.18 (Snellen acuity)
Bielory et al. [14]	2	1	intracameral lidocaine HCl 1% and phenylephrine 2.5% inadvertently preserved with 10% benzalkonium chloride.	Acute corneal decompensation (3)	Medical treatment only (1), Corneal transplantation for decompensated cornea (2)	BCVA = 20/20 (1), NA (1)
Althomali [38]	15	1–2	OVD	Corneal edema (15), hypopyon (8)	Medical treatment only	BCVA: count finger (2) (other retina pathology), BCVA: 20/70 (2), BCVA≥20/50 (11)
Koban et al. [16]	1	1	Inadvertent overdose of intracameral gentamicin	Hyphema, corneal edema, chemosis, hemorrhagic fibrinous reaction, Corneal decompensation	Penetrating keratoplasty after medical treatment	BCVA: 20/60
Cetinkaya et al. [54]	5	1	unknown	corneal edema (5), anterior chamber reaction (5), fibrin (3), hypopyon (3), increased intraocular pressure (3)	Penetrating keratoplasty (2)	BCVA: 20/100 (1), BCVA: 20/40 (1), BCVA≥20/30 (3)
Ari et al. [27]	19 (pediatric patients)	1–2	Ethylene oxide gas sterilization	Corneal edema, anterior chamber reaction,	Medical treatment only (18), Penetrating keratoplasty (1)	NA
Buzard et al. [15]	2	1	Generic trypan blue	Cornea edema, anterior chamber reaction, hypopyon	Penetrating keratoplasty (2)	NA
Maier et al. [5]	24	1–2	Contamination of corneal trephine	Graft infiltration, corneal stromal edema	Medical treatment only (24)	NA
Choi et al. [28]	15	NA	Ethylene oxide gas sterilization	Corneal edema, anterior chamber reaction, conjunctival injection, pupil irregularity, fibrin reaction	Penetrating keratoplasty (5)	BCVA≥20/200 (14), Light perception (1),

*IOL* intraocular lens, *BCVA* best corrected visual acuity, *NA* not available, *n* case number

**Table 2:** Clinical manifestation of toxic anterior segment syndrome in large-scale outbreak studies

Clinical manifestation	Suzuki et al. [18] (n:251)	Oshika et al. [8] (n:147)	Endophthalmitis vitrectomy study [12] (n:420)
Onset after surgery (day)	38.44 ± 32.29 days Range:0–161	13.1 ± 16.4 days Range: 1–88	6 days Range: 1–63
Pain	NA	9.5%	74.3%
Blurred vision	NA	NA	94.3%
Lid swelling	NA	NA	34.5%
Injection and/or chemosis	41.4%	39.8%	82.1%
Corneal edema	19.1%	15.6%	NA
Anterior chamber fibrin reaction or membrane formation	26.7%	43.1%	NA
Anterior chamber cell and/or flare	99.2%	Cells (97.2%), flare (63.0)	NA
Hypopyon	22.7%	10.6%	85.7%
Keratic precipitates	27.9%	21.6%	NA
Anterior vitreous opacities	21.5%	23.8%	NA
Media opacity	NA	NA	99.5%
Red reflex present	NA	NA	32.0%
Macular edema or other retinal abnormalities	NA	3.8%	NA

*NA* not available

The last column refers clinical manifestation of endophthalmitis for the comparative purpose

Although the onset of symptoms and involvement of vitreous was suggested as differentiating points between TASS and infectious endophthalmitis in some studies, the time before the onset of TASS is now known to vary widely [1, 8, 18]. TASS typically starts earlier (within 24 h after surgery) than infectious endophthalmitis (4–7 days after surgery). However, later onset cases are not rare. Miyake et al. reported 6 cases of late-onset TASS occurring 42 to 137 days after surgery [19]. In cases of TASS related to intraocular lens (IOL) contamination, the mean onset time from surgery to TASS was approximately 38 days [18].

Even after successful treatment, eyes with TASS can suffer significant sequelae. Avisar et al. investigated the endothelial morphology of eyes after TASS and found lower cell density, higher cell area and lower percentage of hexagonal cells [20].

Clinicians should be aware that the typical signs of TASS can be masked by strong topical steroids during the early postoperative period. Thus in some cases, TASS can manifest after discontinuation of topical steroids [21].

### Etiology

Investigating the causative agent of TASS is difficult and sometimes unsuccessful. In many cases, the exact cause of TASS remains unknown even after a thorough investigation [7, 9]. Sengupta et al. reported that the etiology was not found even after a careful search in approximately 51.7% of TASS cases in their large case series (60 cases after uneventful cataract surgery) [7]. To date, the major causes implicated in TASS include inadequate cleaning of surgical instruments, contamination of surgical instrument or IOLs, and adverse drug reactions [1, 22, 23].

#### Surgical instrument contamination

The American Society of Cataract and Refractive Surgery (ASCRS) TASS Task Force suggested that improper cleaning of surgical instruments is the most common cause of TASS [2, 22, 24]. Inadequate flushing of hand pieces, the use of enzymatic detergents and the use of ultrasound baths were the most common factors involved in TASS, especially enzymatic detergents for cleaning instrument containing endotoxins, which are not deactivated by autoclave sterilization [1, 23, 24]. It is noteworthy that enzyme remnants still exist at the tip of surgical instruments even after vigorous flushing and rinsing [25]. These enzymes are not inactivated by heat of less than 140 °C and most Statim™ (SciCan, Canonsburg, PA) autoclaves reach temperatures of only 138 °C [26]. The dose-dependent toxicity of enzymatic detergents in corneal endothelium has previously been verified in animal models [26]. Therefore, the ASCRS Task Force on Ophthalmic Instrument Cleaning and Sterilization recommended avoiding the use of enzymatic detergents for ophthalmic instrument cleaning [24]. Additionally, ethylene oxide gas sterilization of surgical tubing lines resulted in severe TASS in 13 and 15 patients, respectively [27, 28]. Moreover, bacterial biofilm contamination of autoclave reservoirs can produce heat stable bacterial toxins continuously and contaminate surgical instruments during autoclaving [10].

#### Intracameral injection

Corneal endothelial toxicity and TASS are potential concerns following the intracameral injection of any pharmacologic agents. Drug components, inadvertent dilution with causative agents, preservatives, abnormal pH, or increased osmolality are all possible causes of TASS [29]. In addition, Lockington et al. found free radicals present in 19 commonly used intracameral drug preparations including phenylephrine, cefuroxime, lidocaine and bevacizumab [30]. These free radicals can induce a dose dependent cellular damage. Previously, the inadvertent use of a balanced salt solution with a low pH of 6.0 resulted in 12 cases of TASS in an outbreak [7]. Recently, Bielory et al. reported that the inadvertent intracameral injection of lidocaine HCl 1% and phenylephrine 2.5% preserved with 10% benzalkonium chloride resulted in severe TASS with irreversible corneal decompensation [14]. Koban et al. reported that inadvertent intracameral injection of a high dose (20 mg/0.5 ml) of gentamicin, prepared for subconjunctival injection, induced severe TASS and bullous keratopathy [16]. It is also possible that small amounts of gentamicin can access the anterior chamber through surgical incisions after subconjunctival placement [17]. Although it is debated, TASS after intracameral injection of cefuroxime has also been reported [31, 32]. Balanced salt solution (BSS) contamination can be another risk factor for TASS. Andonegui et al. reported five cases of TASS after using BSS prepared in a hospital pharmacy [33]. Inadvertent seeping of ophthalmic ointment into the anterior chamber has also been implicated in causing TASS [34].

#### Indocyanine green dye and trypan blue for lens capsule staining

Anterior lens capsule staining with dyes such as indocyanine green or trypan blue has generally been accepted as a safe and effective method to improve the visualization during capsulorhexis [35]. However, dye agents used for anterior capsule staining can become contaminated during the manufacturing process. Matsou et al. reported five cases of TASS and Buzard et al. reported two cases after using a generic trypan blue for capsule staining [15, 36]. Tandogan et al. investigated the toxic effect of indocyanin green in the anterior chamber of a rabbit [37]. Higher concentrations and longer exposure times have been thought to result in severe inflammation mimicking TASS.

#### Ophthalmic viscosurgical devices

Contamination or denaturation of ophthalmic viscosurgical devices (OVDs) can be a potential cause of TASS. Suspicious batches of OVDs evoked 17 and 15 cases of TASS in separate studies [7, 38]. Contamination by endotoxin during manufacturing was suspected in cases where the OVDs were derived from bacterial fermentation. Thus the need for guidelines for endotoxin limits in ophthalmic preparations has been proposed [38, 39].

.Traces of OVD residue attached to surgical instruments are sometimes not removed completely during cleaning and can denature to become toxic material. OVD denaturation can occur due to inappropriate handling during shipping and storage. Recently, another large outbreak (34 cases in 2 weeks) of TASS, possibly related to OVD, was reported [40]. In this case series, utilization of new OVDs prevented further occurrence of TASS.

#### Intraocular lens contamination

Recently heavy metal, such as aluminum, contamination during manufacturing of IOLs was proposed as a possible cause of massive (147 cases) outbreak of subacute TASS in Japan [8]. In another report, 251 cases of late onset TASS were related to a particular type of IOL [18]. Other anecdotal cases of TASS related to IOLs have also been reported [19, 41].

#### Patient’s clinical characteristics

Patients’ factors can also contribute to TASS development. Yazqan et al. investigated the systemic disease profile in TASS patients compared to controls. They found that type 2 diabetes mellitus, systemic hypertension, hyperlipidemia, chronic ischemic heart disease, and chronic renal failure were significantly more common in TASS patients [42].

### Evaluation for etiology

**Fig. 2:**
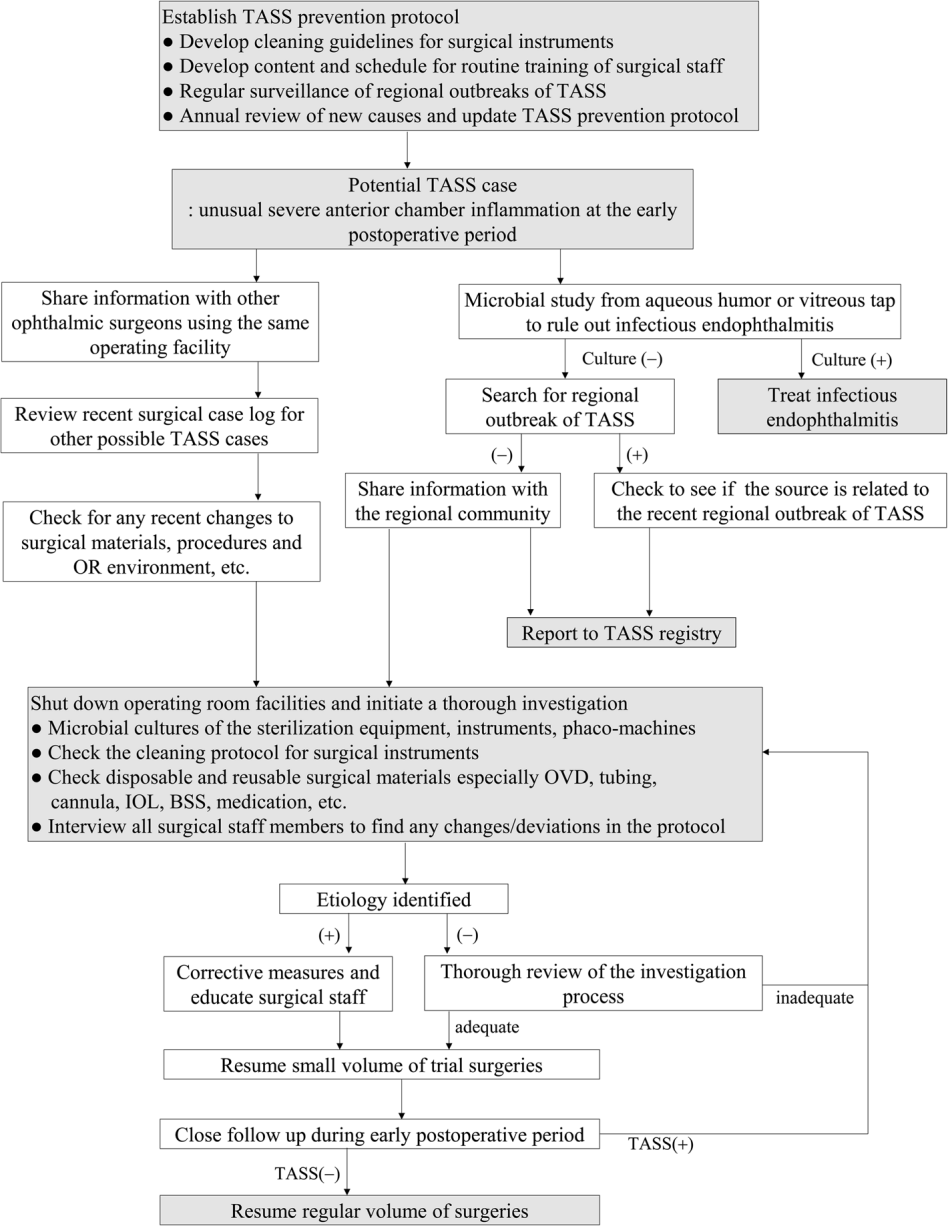
Sample algorithm for the prevention and investigation of TASS

In cases of a TASS outbreak in a single institution, it is recommended that the surgical facility halts operations and immediately initiate a thorough investigation. It is important to share suspected TASS cases with other ophthalmic surgeons using the same surgical facility. By doing this, the incidence can be contained. It is also recommended that outbreaks be announced to outside surgeons in the same or different regions to share information and find any possible clues to explain regional outbreaks originating from IOLs or OVDs. All surgical staff members should be interviewed and any purposeful or inadvertent changes to their protocol should be investigated as shown in Fig. 2 [9].

For laboratory evaluation, aqueous tapping for microbial studies is recommended. Especially in severe cases of TASS, differentiating between bacterial endophthalmitis and TASS is a critical step in the treatment algorithm. Vitreous involvement is more common in bacterial endophthalmitis and, in this case, vitreous tapping is also necessary. However, Gram stain and culture are often negative in some infectious endophthalmitis [43]. While waiting for microbial culture and sensitivity results, any recent changes in the surgical setup such IOLs, OVDs, solutions, surgical drapes, latex gloves, moving to a new operating room, using a new sterilization system or new phacoemulsification platform should be investigated as possible sources of contamination. Consulting infection prevention teams inside the hospital can be helpful. Bacterial culture screening of operating rooms, operating tables and surgical microscopes can provide important additional information after positive microbial test results. Obtaining cultures from sterilization systems is also essential for ruling out any contamination by heat stable bacterial toxins.

Suspected TASS cases should be reported to the TASS Registry (www.ascrs.org/tass-registry) run by the ASCRS. The website provides useful information such as TASS guidelines, a free link to the “Instrument Re-Processing and Product Questionnaire” (www.tassregistry.org/tass-combined-survey.cfm) developed by the ASCRS, and the quick link for the voluntary reporting of TASS to the Food and Drug Administration (FDA).

### Prevention

The most important step for preventing TASS is raising awareness. It is very helpful to establish TASS prevention protocols and regularly train surgical staff. In the event of a TASS outbreak, this established protocol can be a valuable guideline to determine possible etiologies.

The use of preservative-free medications is important as well. The instillation of ophthalmic ointment after cataract surgery has largely been abandoned due to the risk of TASS by inadvertent entry into the anterior chamber.

Adequate cleaning and sterilization of ophthalmic surgical instruments are crucial to prevent TASS. The ASCRS, American Academy of Ophthalmology and American Society of Ophthalmic Registered Nurses participated in the joint TASS Task Force and published guidelines on how to clean and sterilize intraocular surgical instruments to prevent TASS [44]. (Table 3) Switching to single-use disposable instruments is also an effective way to prevent contaminating instruments during cleaning and sterilization.

**Table 3:** Recommendations for cleaning and sterilizing intraocular surgical instruments modified from the guideline proposed by ASCRS, AAO and ASORN [44]

Ensure adequate time for thorough cleaning and sterilization of instrument	
•Rigorous adherence to recommended procedures for cleaning and sterilization •Sufficient inventory of instruments to meet surgical volume and to provide adequate time for cleaning and sterilization	
Follow manufacturer’s directions for use for cleaning and sterilization	
Ophthalmic viscosurgical device solutions should not be allowed to dry on instruments	
•Instruments should be rinsed with sterile water immediately following the use	
Used instruments should be transported from the operating room in a closed container to the decontamination area	
Whenever possible, use disposable instruments and/or tubing and then discard after each use.	
•Do not reuse devices labeled for single use only.	
Clean intraocular instruments separately from non-intraocular surgical instruments.	
Avoid using enzymatic detergents for the cleaning of intraocular instruments.	
•When the use of enzymatic detergents is necessary, instruments should be thoroughly rinsed with copious volumes of water to remove all detergent.	
Ultrasonic cleaners should be emptied, cleaned, disinfected, rinsed and dried at least daily and preferably after each use.	
Do not reuse manual cleaning tools unless designed for reuse.	
•If brushes are reused, they should be designed for reuse and cleaned and treated with high-level disinfection or sterilization, preferably after each use, or at least once daily.	
Rinsing should provide flow of water through or over instruments and agitation in a basin of water should not be used.	
•Following thorough rinsing, instruments with lumens should be dried with forced or compressed air.	
If reusable woven materials are used for draping or wrapping trays or instruments, they should be laundered thoroughly between each use to eliminate surgical compounds, debris, and cleaning agents.	
Cleanliness and integrity of instruments should be verified.	
Sterilization	
•Glutaraldehyde is not recommended because of the toxicity of glutaraldehyde residues. •Low temperature methods of sterilization should not be used unless validated by the instrument manufacturer. •Regular autoclave sterilizers are preferred over Statim™ sterilizers because higher temperatures up to 190 °C can be reached. •Verification of sterilizer function should be completed at least weekly, preferably daily.	
Have a written policy in place for protocols for what happens to the instruments prior to and after each case in accordance with the manufacturer’s instructions.	

### Treatment of TASS

Topical steroids are the mainstay treatment of TASS. In mild cases of TASS, frequent instillation (4 to 8 times per day) of a potent steroid, particularly 1% prednisolone acetate or alternatively dexamethasone 0.1% can be the initial choice of treatment [5, 9, 27, 36, 38]. Subconjunctival dexamethasone injection can be used when the effect of topical steroid is limited. In cases of severe TASS with dense fibrin and hypopyon, oral prednisolone up to 40 mg per day can be necessary to control the inflammation [19]. A topical NSAID can be added for pain control. Microbial culture can be negative in up to 30% of bacterial endophthalmitis [45]. Therefore, the combined use of broad spectrum antibiotics such as moxifloxacin is recommended, especially when the severe inflammation hinders the discrimination between TASS and bacterial endophthalmitis. In patients with severe fibrin reaction which is refractory to conventional steroid treatment, intracameral injection of recombinant tissue type plasminogen activator (25 μg/0.1 ml) can be effective [46]. Close follow up of the patient is critical to ensure that the inflammation responds to treatment. In cases where inflammation worsens with treatment, a repeat culture is recommended to rule out missed infectious endophthalmitis. Surgical interventions such as anterior chamber washout, vitrectomy or IOL removal can be performed according to the surgeon’s discretion, especially if the inflammation persist despite adequate medical treatment [8]. Ohika et al. and Suzuki et al. reported 29.3% and 43.4% of TASS cases, respectively, required surgical intervention such as anterior chamber irrigation, anterior vitrectomy, vitrectomy and IOL removal in their large case series [8, 18].

Mild cases of TASS usually resolve without any complications. However, irreversible corneal endothelial damage and decompensation by uncontrolled severe TASS may require corneal transplantation. Endothelial keratoplasty is an effective way to replace decompensated corneal endothelium after severe TASS [47, 48]. Kaur et al. reported that the time interval between TASS and endothelial keratoplasty is critical for successful surgical outcomes [49]. In their report, a time interval of less than 3 months (3cases) resulted in high rate of graft failure, while 12 cases with time intervals greater than 3 months resulted in 100% successful outcomes. Therefore, the surgeon should be prudent in deciding the timing of endothelial keratoplasty. In cases of secondary glaucoma following TASS, anti-glaucoma medications and, sometimes, glaucoma surgery is needed [28, 34]. Cystoid macular edema can occur due to TASS and this may require intraocular steroids or anti-VEGF injection treatment [13].

### Visual outcome

As expected, prompt diagnosis and initiation of appropriate treatment determine the visual prognosis of TASS. By quickly resolving the diagnosis and treatment, irreversible damage to the corneal endothelium, trabecular meshwork and macula can be minimized. Visual outcomes of TASS seem to be relatively good with appropriate treatment [7, 8, 18]. Sengupta et al. reported that 58 out of 60 TASS eyes achieved a best corrected visual acuity of 6/9 or better on 1 month after treatment; however, significant numbers of eyes were complicated by atrophic iris (24%), posterior capsule opacification (16%), severe anterior capsular phimosis (12.5%) and cystoid macular edema (4%) 6 months after treatment [7]. Suzuki et al. reported the visual prognosis of all patients with IOL- related TASS was good with no single case of severe visual deterioration [18]. Oshika et al. reported only 2 out of 201 TASS cases resulted in best corrected visual acuity deterioration to 20/50 and 20/100, respectively, and those were due to macular edema after TASS treatment [8].

However, it is likely that the visual outcome after TASS treatment is dependent on the etiology. TASS caused by inadvertent intracameral drug injection may result in irreversible corneal damage and poor visual prognosis. TASS related to the exposure of an intraocular instrument to glutaraldehyde (2%) resulted in irreversible corneal decompensation in 100% of affected eyes [50]. Accidental use of methylene blue for capsule staining and accidental intracameral entry of gentamicin also resulted in irreversible corneal decompensation [16, 51, 52]. Bielory et al. reported 2 out of 3 TASS cases related to the inadvertent injection of 10% benzalkonium chloride containing medication needed corneal transplantation [14]. Werner et al. reported 3 out of 8 TASS cases caused by inadvertent seeping of antibiotic-steroid ointment into the anterior chamber resulted in corneal decompensation [34].

Some TASS cases caused by gas sterilization also showed poor visual outcomes. Choi et al. and Smith et al. reported 5 out of 15 TASS cases related to ethylene oxide gas sterilization and 6 out of 10 related to plasma gas sterilization required penetrating keratoplasty due to corneal decompensation, respectively [28, 53].

## Conclusions

Albeit rare, TASS can perplex ophthalmic surgeons and result in unfortunate outcomes. Whenever TASS is suspected, a thorough investigation of possible etiologies is critical, as is sharing the information with colleagues. TASS can occur anytime and unexpectedly. However, anterior segment surgeons should be aware that TASS is mostly preventable by the establishment of TASS prevention protocols, regular training, and thorough adherence to recommendations for cleaning and sterilizing intraocular surgical instruments.

## Abbreviations

BSS: Balanced salt solution
IOL: Intraocular lens
OVD: Ophthalmic viscoelastic device
TASS: Toxic anterior segment syndrome
